# Supplementary material for: The Association of Age, Sex, and BMI on Lower Limb Neuromuscular and Muscle Mechanical Function in People with Multiple Sclerosis
Source: Biomedicines. 2024 Apr 28;12(5):971. doi: 10.3390/biomedicines12050971 (PMC11117965; doi:10.3390/biomedicines12050971)
Supplement: Supplementary file 1 [file biomedicines-12-00971-s001.zip › biomedicines-2916272-supplementary.pdf]

biomedicines

MDPI

Supplementary Table S1: CMJ characteristic in pwMS and HC according to sex, age and BMI

	Sex	AGE							BMI						
				pwMS			HC				pwMS			HC	
**Jump Parameters**	**Sex**	**Age**	**N**	**Mean (±SD)**	**CI 95%**	**N**	**Mean (±SD)**	**CI 95%**	**BMI**	**N**	**Mean (±SD)**	**CI 95%**	**N**	**Mean (±SD)**	**CI 95%**
Braking Time	Male	18-30	12	0.20 ± 0.05	0.16 – 0.23	13	0.17 ± 0.04	0.14 – 0.19	18-25	29	0.20 ± 0.05	0.18 – 0.22	23	0.17 ± 0.04	0.156 – 0.19
		31-49	41	0.21 ± 0.07	0.19 – 0.24	22	0.18 ± 0.06	0.15 – 0.20	25-30	17	0.24 ± 0.07	0.20 – 0.27	11	0.18 ± 0.08	0.12 – 0.23
		50-65	1	0.23	-	4	0.20 ± 0.02	0.17 – 0.23	30+	8	0.20 ± 0.08	0.13 – 0.27	5	0.18 ± 0.04	0.13 – 0.23
	Female	18-30	32	0.21 ± 0.10	0.17 – 0.24	17	0.18 ± 0.07	0.14 – 0.22	18-25	70	0.21 ± 0.09	0.19 – 0.23	44	0.18 ± 0.07	0.16 – 0.21
		31-49	64	0.22 ± 0.10	0.20 – 0.25	35	0.19 ± 0.08	0.16 – 9.22	25-30	26	0.20 ± 0.07	0.17 – 0.23	11	0.20 ± 0.10	0.14 – 0.27
		50-65	14	0.24 ± 0.08	0.18 – 0.28	7	0.24 ± 0.08	0.16 – 0.31	30+	14	0.30 ± 0.12	0.23 – 0.37	4	0.26 ± 0.09	0.12 – 0.39
Propulsive Time	Male	18-30	12	0.32 ± 0.08	0.27 – 0.37	13	0.29 ± 0.06	0.25 – 0.32	18-25	29	0.31 ± 0.06	0.29 – 0.33	23	0.29 ± 0.05	0.27 – 0.32
		31-49	41	0.32 ± 0.11	0.29 – 0.35	22	0.29 ± 0.06	0.27 – 0.32	25-30	17	0.32 ± 0.07	0.28 – 0.35	11	0.28 ± 0.05	0.25 – 0.32
		50-65	1	0.30	-	4	0.32 ± 0.03	0.26 – 0.37	30+	8	0.37 ± 0.22	0.18 - 0.55	5	0.30 ± 0.10	0.18 – 0.42
	Female	18-30	32	0.31 ± 0.08	0.28 – 0.33	17	0.27 ± 0.05	0.25 – 0.30	18-25	70	0.32 ± 0.09	0.29 – 0.34	44	0.27 ± 0.05	0.26 – 0.29
		31-49	64	0.31 ± 0.09	0.29 – 0.33	35	0.28 ± 0.05	0.26 – 0.30	25-30	26	0.30 ± 0.07	0.28 – 0.33	11	0.29 ± 0.07	0.25 – 0.34
		50-65	14	0.33 ± 0.09	0.28 – 0.38	7	0.29 ± 0.06	0.23 – 0.35	30+	14	0.30 ± 0.06	0.27 – 0.33	4	0.30 ± 0.09	0.15 – 0.44
Flight Time	Male	18-30	12	0.38 ± 0.06	0.35 – 0.42	13	0.41 ± 0.05	0.38 – 0.44	18-25	29	0.37 ± 0.04	0.36 – 0.39	23	0.40 ± 0.04	0.38 – 0.42
		31-49	41	0.35 ± 0.05	0.33 – 0.37	22	0.38 ± 0.04	0.36 – 0.40	25-30	17	0.35 ± 0.06	0.31 – 0.38	11	0.38 ± 0.05	0.35 – 0.41
		50-65	1	0.30	-	4	0.36 ± 0.01	0.34 – 0.38	30+	8	0.31 ± 0.04	0.28 – 0.35	5	0.35 ± 0.05	0.29 – 0.41
	Female	18-30	32	0.31 ± 0.05	0.29 – 0.32	17	0.35 ± 0.02	0.34 – 0.36	18-25	70	0.30 ± 0.04	0.29 – 0.31	44	0.33 ± 0.03	0.32 – 0.34
		31-49	64	0.29 ± 0.04	0.28 – 0.30	35	0.32 ± 0.03	0.31 – 0.33	25-30	26	0.28 ± 0.04	0.27 – 0.30	11	0.31 ± 0.04	0.29 – 0.34
		50-65	14	0.25 ± 0.03	0.23 – 0.27	7	0.25 ± 0.03	0.21 – 0.28	30+	14	0.24 ± 0.04	0.22 – 0.26	4	0.23 ± 0.02	0.20 – 0.27
Force at Zero Velocity	Male	18-30	12	14.69 ± 6.19	10.76 – 18.63	13	17.33 ± 7.59	12.74 – 21.92	18-25	29	13.36 ± 5.38	11.31 – 15.40	23	18.37 ± 4.49	16.43 – 20.31
		31-49	41	11.65 ± 5.93	9.78 – 13.52	22	19.02 ± 4.78	16.90 – 21.14	25-30	17	11.53 ± 6.43	8.22 – 14.84	11	19.86 ± 6.95	15.19 – 24.53

		50-65	1	7.36	-	4	15.68 ± 3.91	9.96 – 21.91	30+	8	9.74 ± 7.27	3.66 – 15.82	5	13.10 ± 6.71	4.77 – 21.44
	Female	18-30	32	10.19 ± 4.83	8.45 – 11.93	17	15.77 ± 5.45	12.96 – 18.57	18-25	70	10.94 ± 5.62	9.60 – 12.27	44	15.34 ± 5.05	13.80 – 16.88
		31-49	64	10.47 ± 5.66	9.05 – 11.88	35	14.63 ± 5.44	12.76 – 16.49	25-30	26	10.04 ± 5.27	7.91 – 12.17	11	14.31 ± 6.39	10.02 – 18.61
		50-65	14	10.17 ± 6.19	6.59 – 13.74	7	13.88 ± 5.79	8.52 – 19.23	30+	14	7.98 ± 4.51	5.38 – 10.58	4	11.15 ± 6.59	0.67 – 21.63
Peak Force	Male	18-30	12	20.37 ± 1.93	19.14 – 21.56	13	23.31 ± 3.00	21.49 – 25.12	18-25	29	20.13 ± 1.77	19.46 – 20.80	23	21.72 ± 2.50	20.64 – 22.80
		31-49	41	19.71 ± 1.64	19.19 – 20.22	22	21.78 ± 2.13	20.84 – 22.72	25-30	17	19.24 ± 1.56	18.43 – 20.03	11	23.11 ± 3.15	20.99 – 25.26
		50-65	1	18.17		4	19.00 ± 0.73	17.82 – 20.16	30+	8	19.96 ± 1.66	18.57 – 21.35	5	20.87 ± 1.23	19.35 – 22.34
	Female	18-30	32	19.08 ± 2.05	18.34 – 19.81	17	20.57 ± 1.65	19.72 – 21.42	18-25	70	18.94 ± 1.98	18.47 – 19.42	44	20.06 ± 1.84	19.50 – 20.62
		31-49	64	18.78 ± 1.96	18.29 – 19.27	35	19.88 ± 1.79	19.26 – 20.49	25-30	26	18.48 ± 2.01	17.67 – 19.29	11	19.73 ± 1.57	18.68 – 20.79
		50-65	14	17.72 ± 1.97	16.68 – 18.86	7	18.08 ± 2.19	16.05 – 20.10	30+	14	18.13 ± 2.14	16.89 – 19.37	4	18.08 ± 3.06	13.21 – 22.96
Negative Power	Male	18-30	12	4.29 ± 0.99	3.66 – 4.92	13	5.70 ± 1.37	4.86 – 6.53	18-25	29	4.21 ± 1.10	3.79 – 4.63	23	5.02 ± 1.23	4.49 – 5.55
		31-49	41	3.87 ± 1.17	3.50 – 4.24	22	4.76 ± 1.13	4.26 – 5.26	25-30	17	3.83 ± 0.89	3.38 – 4.29	11	5.42 ± 1.31	4.54 – 6.30
		50-65	1	3.37	-	4	4.25 ± 0.88	2.85 – 5.65	30+	8	3.31 ± 1.50	2.06 – 4.56	5	4.12 ± 1.14	2.70 – 5.54
	Female	18-30	32	3.19 ± 1.11	2.79 – 3.59	17	4.19 ± 1.17	3.58 – 4.79	18-25	70	3.23 ± 1.09	2.97 – 3.49	44	3.92 ± 1.06	3.60 – 4.24
		31-49	64	3.17 ± 0.98	2.92 – 3.41	35	3.86 ± 0.92	3.4 – 4.17	25-30	26	3.06 ± 0.84	2.72 – 3.40	11	3.93 ± 0.89	3.33 – 4.53
		50-65	14	2.38 ± 0.98	1.81 – 2.94	7	3.00 ± 0.80	2.26 – 3.75	30+	14	2.32 ± 0.82	1.84 – 2.78	4	2.85 ± 0.63	1.84 – 3.85
Positive Power	Male	18-30	12	18.17 ± 3.49	15.95 – 20.38	13	19.54 ± 3.97	17.13 – 21.93	18-25	29	17.63 ± 2.83	16.56 – 18.70	23	18.78 ± 2.76	17.59 – 19.97
		31-49	41	16.19 ± 3.42	15.11 – 17.28	22	17.79 ± 2.41	16.72 – 18.86	25-30	17	16.07 ± 3.91	14.06 – 18.08	11	17.84 ± 2.95	15.85 – 19.82
		50-65	1	12.79	-	4	16.00 ± 0.38	15.40 – 16.60	30+	8	13.79 ± 3.52	10.84 – 16.72	5	16.27 ± 4.38	10.84 – 21.70
	Female	18-30	32	14.47 ± 3.14	13.34 – 15.60	17	15.96 ± 0	15.23 – 16.69	18-25	70	14.09 ± 2.52	13.48 – 14.69	44	15.04 ± 2.39	14.32 – 15.77
		31-49	64	13.63 ± 2.28	13.06 – 14.20	35	14.56 ± 2.46	13.72 – 15.40	25-30	26	13.59 ± 2.55	12.56 – 14.61	11	14.12 ± 1.98	12.79 – 15.45
		50-65	14	11.43 ± 2.29	10.11 – 12.75	7	11.22 ± 1.53	9.80 – 12.63	30+	14	11.15 ± 2.64	9.62 – 12.67	4	10.57 ± 1.06	8.88 – 12.26
Jump Height	Male	18-30	12	18.52 ± 5.27	15.17 – 21.87	13	21.21 ± 4.82	18.29 – 24.12	18-25	29	17.49 ± 4.01	15.96 – 19.00	23	19.77 ± 4.41	17.86 – 21.68
		31-49	41	15.40 ± 4.20	14.08 – 16.73	22	17.84 ± 4.19	15.98 - 19.70	25-30	17	15.22 ± 5.15	12.57 – 17.87	11	18.22 ± 4.39	15.27 – 21.17
		50-65	1	10.80	-	4	15.76 ± 1.09	14.03 – 17.49	30+	8	12.33 ± 3.20	9.66 – 15.01	5	15.20 ± 4.27	9.90 – 20.51
	Female	18-30	32	11.84 ± 3.55	10.56 – 13.11	17	14.82 ± 1.96	13.81 – 15.83	18-25	70	11.47 ± 2.70	10.83 – 12.11	44	13.45 ± 2.78	12.61 – 14.30
		31-49	64	10.64 ± 2.61	9.98 – 11.29	35	12.88 ± 2.56	12.00 – 13.76	25-30	26	10.18 ± 3.10	8.93 – 11.43	11	12.38 ± 2.75	10.54 – 14.23
		50-65	14	7.77 ± 1.99	6.62 – 8.91	7	7.50 ± 2.15	5.51 – 9.48	30+	14	7.23 ± 2.38	5.86 – 8.60	4	6.77 ± 1.25	4.78 – 8.76

FTCTR	Male	18-30	12	0.49 ± 0.12	0.41 – 0.56	13	0.58 ± 0.11	0.52 – 0.65	18-25	29	0.49 ± 0.09	0.45 – 0.52	23	0.54 ± 0.10	0.49 – 0.58
		31-49	41	0.46 ± 0.09	0.43 – 0.49	22	0.52 ± 0.08	0.48 – 0.56	25-30	17	0.43 ± 0.10	0.38 – 0.48	11	0.54 ± 0.08	0.49 – 0.59
		50-65	1	0.35	-	4	0.43 ± 0.03	0.38 – 0.48	30+	8	0.43 ± 0.09	0.35 – 0.51	5	0.49 ± 0.13	0.33 – 0.65
	Female	18-30	32	0.44 ± 0.12	0.40 – 0.48	17	0.51 ± 0.08	0.47 – 0.55	18-25	70	0.43 ± 0.10	0.40 – 0.45	44	0.48 ± 0.09	0.45 – 0.51
		31-49	64	0.41 ± 0.10	0.38 – 0.43	35	0.47 ± 0.09	0.44 – 0.50	25-30	26	0.40 ± 0.10	0.36 – 0.44	11	0.45 ± 0.07	0.40 – 0.50
		50-65	14	0.34 ± 0.09	0.29 – 0.39	7	0.35 ± 0.08	0.28 – 0.42	30+	14	0.35 ± 0.10	0.29 – 0.40	4	0.33 ± 0.09	0.19 – 0.47
RSI	Male	18-30	12	2.28 ± 0.89	1.72 – 2.84	13	3.00 ± 0.76	2.54 – 3.46	18-25	29	2.24 ± 0.65	1.99 – 2.48	23	2.65 ± 0.78	2.31 – 2.99
		31-49	41	1.94 ± 0.60	1.75 – 2.13	22	2.41 ± 0.65	2.12 – 2.70	25-30	17	1.80 ± 0.68	1.44 – 2.14	11	2.54 ± 0.61	2.13 – 2.95
		50-65	1	1.29	-	4	1.89 ± 0.10	1.73 – 2.05	30+	8	1.62 ± 0.58	1.14 – 2.11	5	2.15 ± 0.81	1.15 – 3.15
	Female	18-30	32	1.66 ± 0.65	1.42 – 1.89	17	2.09 ± 0.49	1.84 – 2.34	18-25	70	1.57 ± 0.55	1.43 – 1.70	44	1.93 ± 0.58	1.75 – 2.10
		31-49	64	1.45 ± 0.50	1.32 – 1.57	35	1.85 ± 0.54	1.66 – 2.03	25-30	26	1.40 ± 0.54	1.18 – 1.61	11	1.74 ± 0.41	1.46 – 2.01
		50-65	14	1.03 ± 0.41	0.80 – 1.27	7	1.02 ± 0.37	0.68 – 1.36	30+	14	1.00 ± 0.46	0.74 – 1.27	4	0.88 ± 0.27	0.45 – 1.31

Abbreviation: FTCTR = Flight-Time Contraction-Time-Ratio, RSI = Reactive-Strength-Index

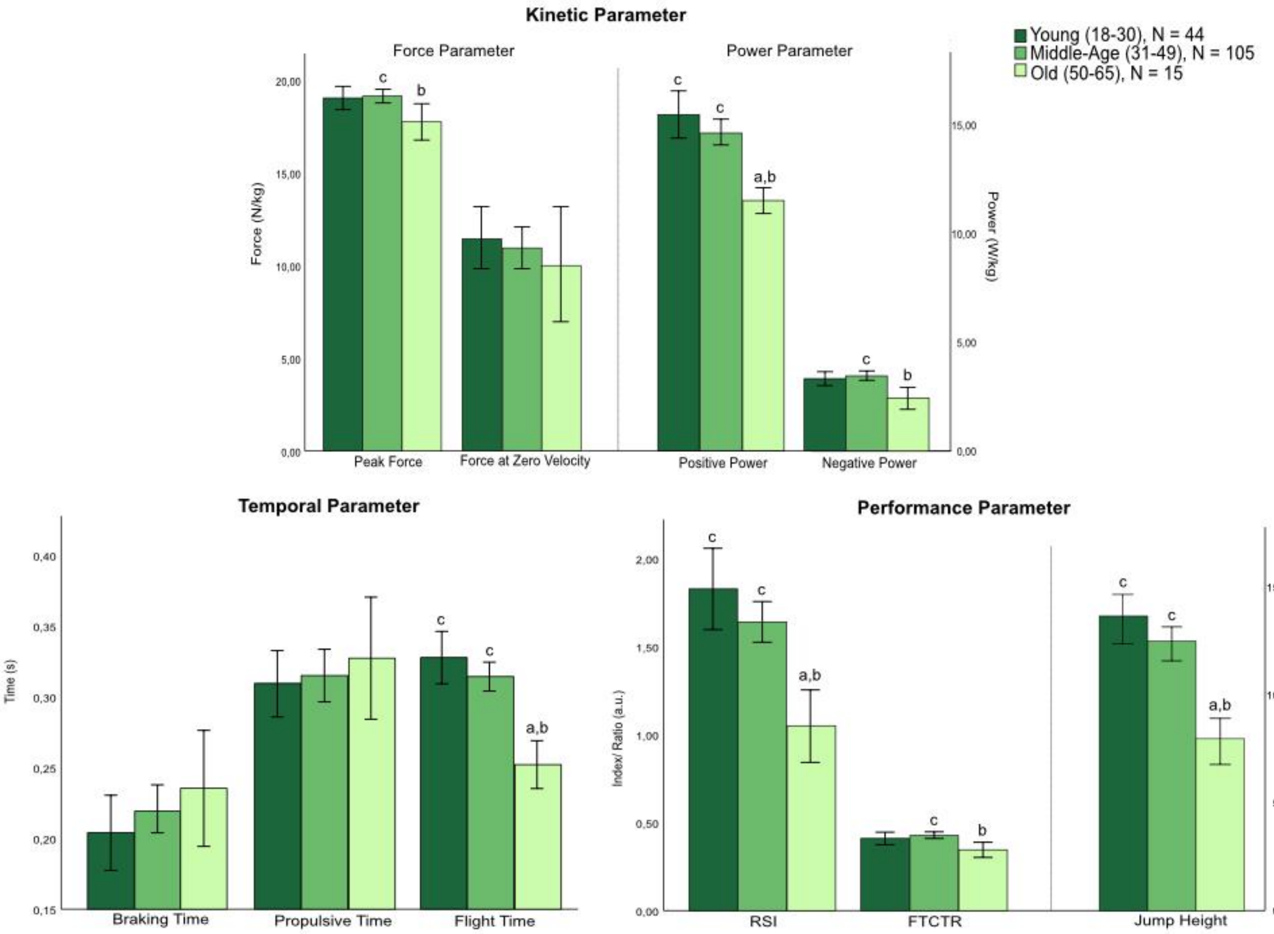

Figure S1. Association between age and the jumping parameters (means ± SD) in pwMS (N = 164) based on the pairwise comparisons tests with adjustments by Bonferroni correction (model 2). Abbreviation: a = significant difference to young ($p < 0.05$). b = significant difference to middle-age ($p < 0.05$). c = significant difference to old ($p < 0.05$). RSI = Reactive-Strength-Index, FTCTR = Flight-Time-Contraction-Time-Ratio, a.u. = arbitrary unit.

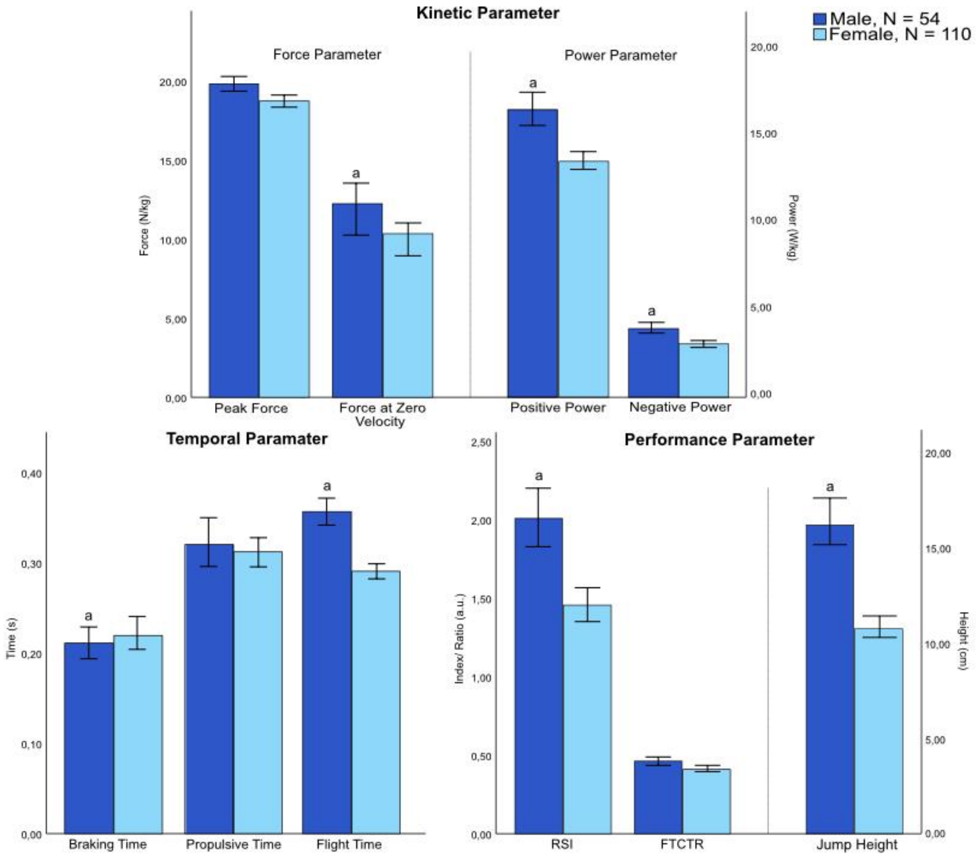

Figure S2. Association between sex and the jumping parameters (means ± SD) for pwMS (N = 164) based on the pairwise comparisons tests with adjustments by Bonferroni correction (model 3). Abbreviation: a = significant difference with female ($p < 0.05$). RSI = Reactive-Strength-Index, FTCTR = Flight-Time-Contraction-Time-Ratio, a.u. = arbitrary unit.

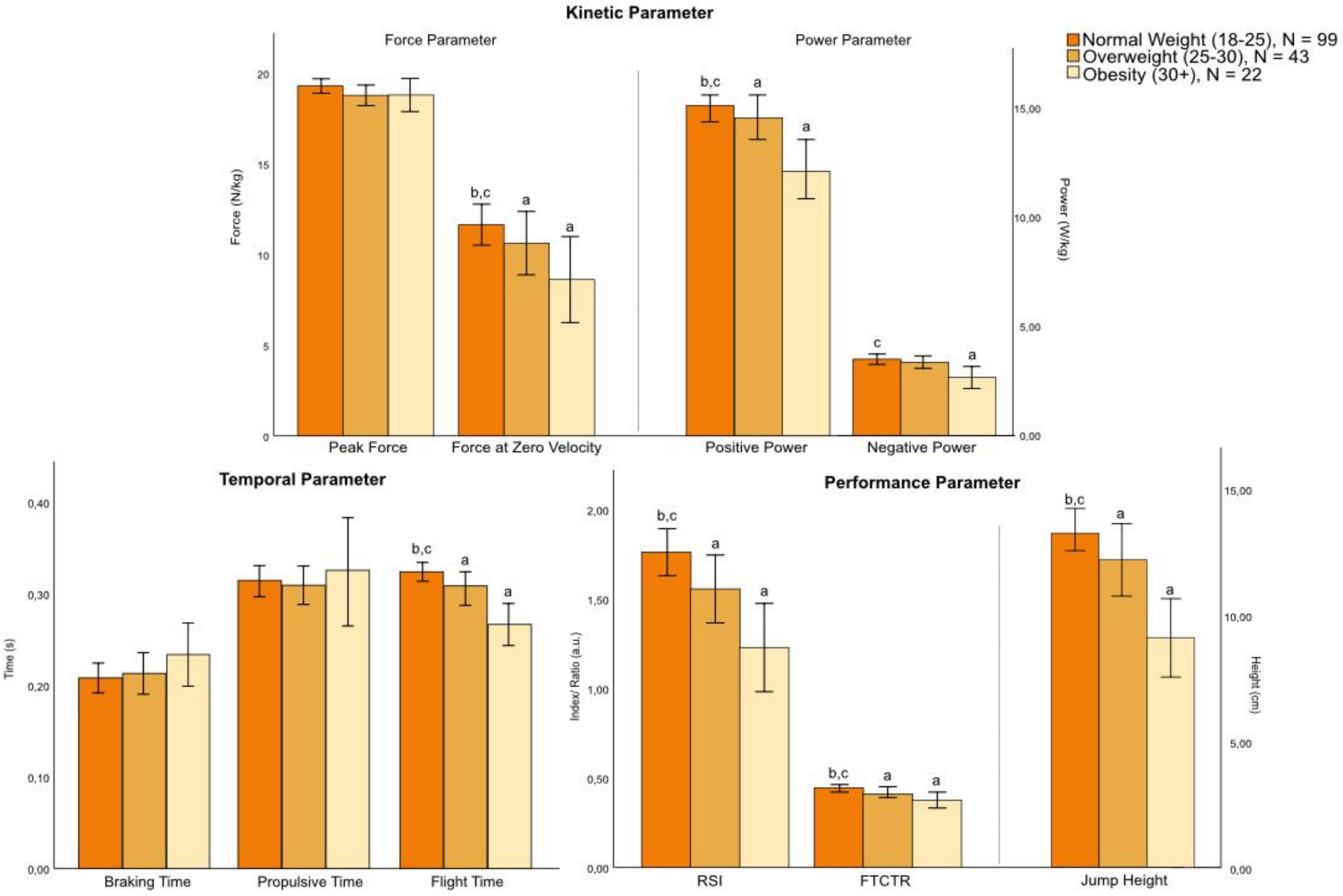

Figure S3. Association between BMI and the jumping parameters (means ± SD) for pwMS (N = 164) based on the pairwise comparisons tests with adjustments by Bonferroni correction (model 4). Abbreviation: a = significant difference to normal weight ($p < 0.05$). b = significant difference to overweight ($p < 0.05$). c = significant difference to obesity ($p < 0.05$). RSI = Reactive-Strength-Index, FTCTR = Flight-Time-Contraction-Time-Ratio, a.u. = arbitrary unit.
